# Catalase ameliorates diabetes‐induced cardiac injury through reduced p65/RelA‐ mediated transcription of *BECN1*

**DOI:** 10.1111/jcmm.13252

**Published:** 2017-06-23

**Authors:** Xu Wang, Youli Tao, Yewei Huang, Kungao Zhan, Mei Xue, Ying Wang, Dandan Ruan, Yangzhi Liang, Xiaozhong Huang, Jianjun Lin, Zhiwei Chen, Lingchun Lv, Santie Li, Gen Chen, Yang Wang, Ruijie Chen, Weitao Cong, Litai Jin

**Affiliations:** ^1^ School of Pharmaceutical Science Wenzhou Medical University Wenzhou China; ^2^ The Ningbo Medical Centre Li Huili Hospital Ningbo China; ^3^ The Second Affiliated Hospital of Wenzhou Medical University Wenzhou China; ^4^ The Health Examination Center the 117th Hospital of Chinese People's Liberation Army Hangzhou China; ^5^ The Affiliated Xiangshan Hospital of Wenzhou Medical University Ningbo Zhejiang China; ^6^ The Fifth Affiliated Hospital of Wenzhou Medical University Lishui China; ^7^ Department of Histology and Embryology Institute of Neuroscience Wenzhou Medical University Wenzhou China

**Keywords:** Catalase, Autophagy, NF‐κB, Diabetes, Cardiomyopathy

## Abstract

Catalase is an antioxidative enzyme that converts hydrogen peroxide (H_2_O_2_) produced by superoxide dismutase from highly reactive superoxide (O_2_^−^) to water and oxygen molecules. Although recent findings demonstrate that catalase, autophagy and the nuclear factor κB (NF‐κB) signalling pathway are centrally involved in diabetic cardiomyopathy (DCM), the interplay between the three has not been fully characterized. Thus, the mechanism responsible for catalase‐mediated protection against heart injury in diabetic mice was investigated in this study, as well as the role of NF‐κB‐p65 in the regulation of autophagic flux was investigated in this study. Western blot analysis revealed that catalase inhibited NF‐κB activity and decreased LC3‐II (microtubule‐associated protein 1 light chain 3) and beclin‐1 (Atg6) expression. Furthermore, up‐regulation of autophagy was detrimental for cardiac function in diabetic mice. Catalase overexpression reduced the level of NF‐κB subunit in the nucleus, where it initiates autophagy through activation of the key autophagy gene *BECN1*. To evaluate the role of the NF‐κB pathway in diabetes‐induced autophagy, Bay11‐7082, an NF‐κB inhibitor, was injected into diabetic mice, which suppressed NF‐κB and attenuated diabetes‐induced autophagy and myocardial apoptosis. In agreement with the *in vivo* results, Bay11‐7082 also inhibited high‐glucose‐induced activation of NF‐κB and the up‐regulation of LC3‐II and beclin‐1 expression in H9c2 cells. In addition, high‐glucose‐induced activation of autophagic flux and apoptosis were largely attenuated by p65 siRNA, suggesting that catalase ameliorates diabetes‐induced autophagy, at least in part by increasing the activity of the NF‐κB pathway and p65‐mediated transcription of BECN1.

## Introduction

DCM is the primary cause of heart failure in patients with diabetes mellitus and is in part responsible for the recent increases in morbidity and mortality from heart disease 1. Hyperglycaemia‐induced impairments in redox balance are considered to be a key trigger for the development of diabetic complications, mediated through increased generation of reactive oxygen species (ROS) and reactive nitrogen species (RNS) 2, 3. The diabetic heart is susceptible to oxidative damage, which can lead to cellular injury, including oxidation of lipids and DNA, and mitochondrial damage 4, 5. In addition, however, the ROS produced by mitochondria is a critical factor in stress‐induced autophagy 6, 7.

Macroautophagy (henceforth referred to as ‘autophagy’) is a highly conserved bulk protein degradation process whereby long‐lived proteins and organelles are enclosed by double‐membraned structures called autophagosomes, which are then targeted to lysosomes for degradation and consequent ATP production to maintain cellular homoeostasis 8, 9. However, excessive activation of the autophagic process in the heart leads to the formation of autophagosomes that digest normal and useful proteins, ultimately leading to cell death and cardiac disease 10.

NF‐κB is a ubiquitously expressed family of Rel‐related transcription factors 11. In unstimulated cells, NF‐κB is predominantly bound to inhibitory κB proteins (IκB) in the cytoplasm. However, in the presence of various kinds of cell stress, such as ROS accumulation or increased secretion of inflammatory cytokines, proteasome‐dependent degradation of IκB occurs, permitting the translocation of NF‐κB to the nucleus, where it binds to the promoter region of target genes involved in the control of cellular responses, including autophagy and apoptosis 12, 13, 14. In addition, beclin‐1 has been shown to play a pivotal role in autophagy, where it serves as a binding partner for several proteins (ATG14L, Vps34) that promote autophagy 15. Moreover, the promoter of the murine *BECN1* autophagy gene contains a conserved NF‐κB binding site 16, suggesting that NF‐κB activation and autophagy may be mechanistically linked. However, the precise relationship between NF‐κB and the product of the *BECN1* gene, beclin‐1, has not been characterized in DCM.

Emerging evidence suggests that hyperglycaemia initiates ROS accumulation in diabetic myocardium 17. Catalase is an antioxidant enzyme that specifically catabolizes hydrogen peroxide (H_2_O_2_) and thus can reduce the accumulation of ROS. Our previous study demonstrated that activation of ROS‐dependent NF‐κB signalling was down‐regulated in the hearts of diabetic mice when catalase was overexpressed 2. However, it remains unclear whether catalase can prevent diabetic heart injury simply by reducing ROS accumulation. To clarify this, we aimed to examine the effect of catalase on both diabetes‐associated NF‐κB activation and autophagy and to determine whether there is a mechanistic link between these phenomena. Our results show that overexpression of catalase relieves the dysregulation of autophagy in diabetic mice. In addition, catalase overexpression reduced the translocation of the NF‐κB subunit p65 to the nucleus, where it binds to the promoter region of *BECN1*, thereby inhibiting autophagy. These findings demonstrate for the first time that catalase can prevent diabetes‐induced cardiac damage, at least in part by reducing autophagy *via* inhibition of NF‐κB transactivation of *BECN1*.

## Materials and methods

### Diabetes models

All animal procedures were approved by Animal Care and Use Committee at the University of Wenzhou Medical. Catalase transgenic (CAT‐TG) mice in FVB genetic background, which were generated using cardiomyopathy‐specific alpha myosin heavy (α‐MHC) promoter 18, were provided by Dr. Y. James Kang (Department of Pharmacology and Toxicology, University of Louisville). The colony expansion and the genotype were performed as previously described 18.

After overnight fasting, 2‐month‐old male CAT‐TG mice and FVB mice were administrated a single intraperitoneal injection of streptozotocin (STZ; Sigma‐Aldrich, St. Louis, MO, USA), at a dose of 150 mg/kg bodyweight (BW). It was dissolved in a 0.1 M sodium citrate buffer (pH 4.5). Three days after STZ administration, animals with a mean non‐fasted blood glucose level ≥16.7 mM were determined as diabetes mice, and all others were excluded from the study 19, 20, sodium citrate buffer‐treated mice served as controls.

For the NF‐κB inhibitor experiment, beginning 3 weeks after induction of diabetes, Bay11‐7082 and DMSO solvent were intraperitoneally injected at a dose of 5 mg/kg (Sigma‐Aldrich), three time per week for 5 weeks 21. In addition, FVB mice and CAT‐TG mice were randomly divided into three groups (*n* = 6 in each group), respectively. For autophagy inhibition and activation experiment, 3‐MA (10 mg/kg/week ip) 22 (Selleck, Houston, TX, USA) and rapamycin (2 mg/kg/day ip) 23 (Selleck) were intraperitoneally injected immediately after the induction of diabetes for 8 weeks, respectively. For accumulation of autophagy flux experiment, WT and CAT‐TG diabetic mice were intraperitoneally treated with bafilomycin (0.3 mg/kg) 3 (Sigma‐Aldrich) daily for 2 weeks after the induction of diabetes.

All mice used in this study were anaesthetized *via* 2% isoflurane inhalation until they were not responsive to toe pinching and killed by overdose isoflurane inhalation, and then hearts were excised and weighed.

### Isolation of neonatal rat cardiac myocytes (NRCMs) and treatment

Primary culture of NRCMs was prepared as described 24. Concisely, 1‐ to 2‐ day‐old Sprague‐Dawley rats were killed by decapitating scissors, and neonatal rats’ hearts were promptly separated and washed to remove blood and debris. Tissue was finely minced followed by sequential digestion with 0.08% trypsin buffer. After tissue digested, cells were pre‐plated for 1 hr to remove non‐myocytes and then plated on the new dishes and culture overnight in DMEM/F12 with 10% foetal bovine serum (FBS; Gibco, Thermo Fisher scientific, Waltham, MA, USA) and 1% penicillin/streptomycin.

### Cell cultures and treatment

H9c2 cells were grown in DMEM, which containing 25 mM glucose, 10% foetal bovine serum (FBS; Gibco, Thermo Fisher scientific) and 1% penicillin/streptomycin, and maintained at 37°C in a humidified incubator containing 5% CO_2_. After reaching 60% of density, cells were pre‐treated with 2 μM Bay11‐7082 or DMSO for 10 min. and then incubated with high glucose (33 mM) for 24 hrs.

### GFP‐LC3 adenovirus transfection

Adenovirus vectors carrying GFP and LC3 cDNA and control vectors were constructed by Genechem (Shanghai, China). H9c2 cells were infected with above adenovirus encoding GFP‐fused LC3 as described 24. GFP‐LC3 cells were transfected with lentiviral vectors for 8 hrs, then continue cultured with 25 mM DMEM for 24 hrs, and pre‐treated with 2 μM Bay11‐7082 for 10 min. and then incubated with high glucose (33 mM) for 24 hrs. Fluorescence images were obtained by fluorescent microscopy (Olympus, Tokyo, Japan).

### Small interference RNA (siRNA) transfection

The siRNA for p65 was purchased in Santa Cruz company (California, USA). H9c2 cells were transiently transfected with p65 or control siRNA by use of Lipofectamine 2000 (Invitrogen, California, USA).

### Immunoprecipitation and immunoblot analyses

Heart tissues were homogenized in a lysis buffer (50 mM Tris [Tris (hydroxymethyl) aminomethane]‐HCl, pH 7.4, 1 mM EDTA, 150 mM NaCl, 20 mM DTT, 0.1% NP40) with PBS (0.02% Tween‐20) and 1% protease inhibitor cocktail, and then centrifuged at 15,000 rpm for 15 min. Antibodies were cross‐linked with Dynabeads protein A (Thermo Fisher Scientific) according to the manufacturer's instructions. The lysis buffer was pre‐cleared with immunoglobulin G (IgG) Dynabeads protein A for 10 min. at 4°C before incubation with antibody‐linked Dynabeads overnight at 4°C. The immunoprecipitated Dynabeads complexes were washed three times with PBS (0.02% Tween‐20). Immunoprecipitated complexes were recovered by resuspending the pellets in the loading buffer and were detected by Western blot, and Western blot details are given in Data S1 online.

### Statistical analysis

Statistical calculations were carried out with Prism 5 (GradPad, SanDiego, CA, USA). Data are presented as mean ± S.E.M. Differences between groups were compared by one‐way anova. Comparison between two groups was performed by *t*‐test. *P* < 0.05 was considered statistically significant.

## Results

### Catalase prevents the development of diabetes‐induced myocardial lesions by inhibiting ROS formation

**Figure 1:**
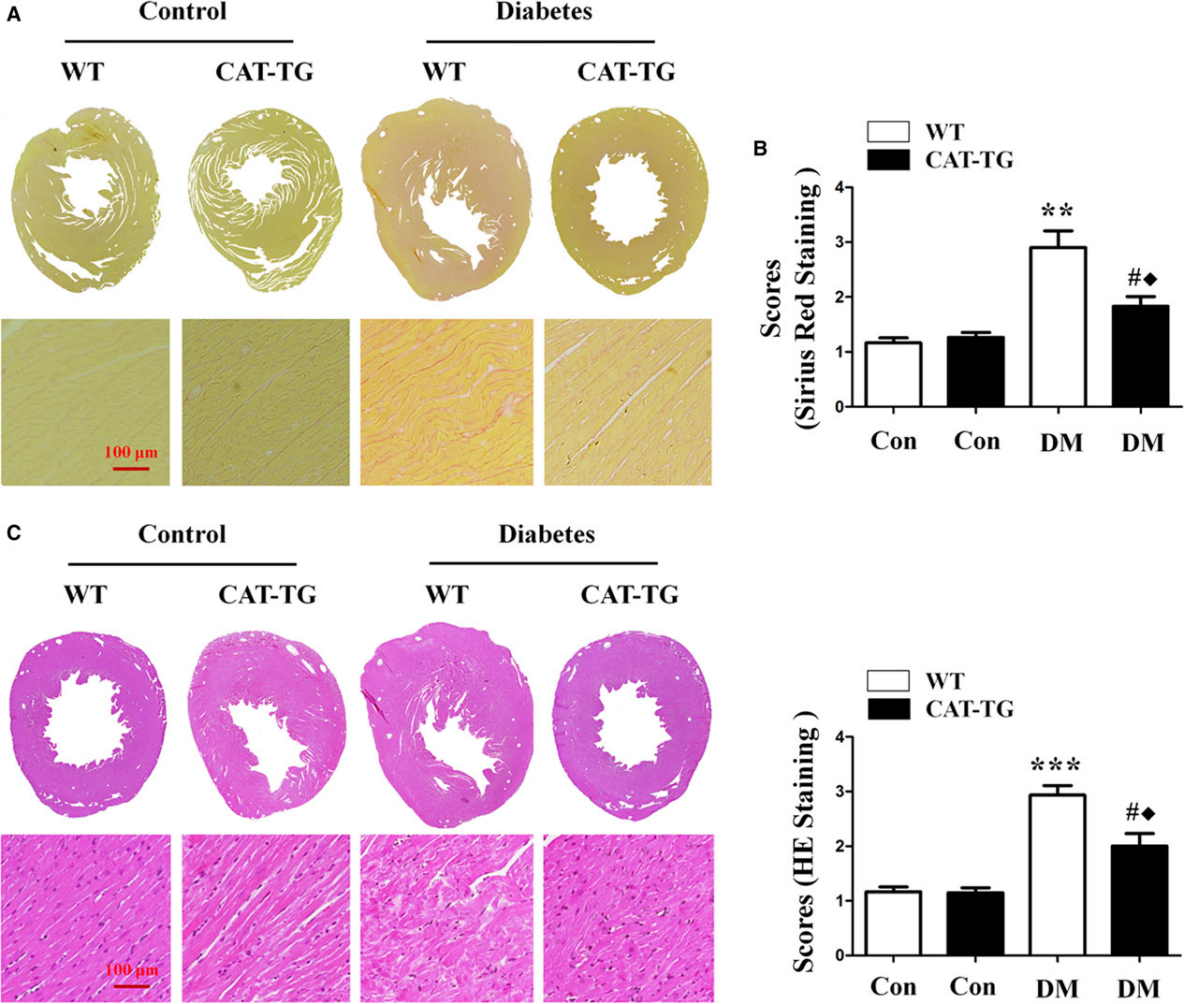
Catalase overexpression alleviates cardiac pathology in diabetic mice. Heart sections in diabetic mice at 8 weeks were stained with Sirius Red (**A**) or Haematoxylin and Eosin (HE) (C), and the condition of the myocardial fibres and the extent of collagen accumulation were assessed under a light microscope. Tissues stained with Sirius Red (**B**) and HE (**D**) were semi‐quantitatively analysed, as described in the Materials and methods. Magnification, ×200; bars = 100 μm. Values are mean ± S.E.M., *n* = 6–8 per group. **P<0.01 and ***P<0.001 *versus* WT Control; #*P*<0.05 *versus* CAT‐TG Control; ◆*P*<0.05 *versus* WT Diabetes.

To evaluate whether catalase has protective effects in diabetic mice, CAT‐TG mice and age‐matched wild‐type (WT) mice were administered STZ, and the blood glucose levels at 0, 2, 4, 8 weeks after induction of diabetes were monitored. The results demonstrated no significant differences in blood glucose levels between WT and CAT‐TG diabetic mice (Fig. S1). Echocardiography (Data S1) was then used to evaluate left ventricular (LV) function in control mice 0, 2, 4 and 8 weeks after the onset of diabetes. This revealed that CAT‐TG mice had lower E/A ratios and left ventricular fractional shortening (LVFS%) than WT diabetic mice. Similarly, the ratios of heart mass to tibial length (HW/TL) and left ventricular mass to body mass (LVM/BW) were significantly lower in CAT‐TG diabetic mice than in WT diabetic mice (Table S1). Sirius red staining of histological sections showed that cardiac‐specific overexpression of catalase dramatically ameliorated the accumulation of collagen (Fig. 1A and B, Fig. S2), and haematoxylin and eosin (HE)‐stained sections demonstrated improved myofibrillar histology, most notably in mice 8 weeks after the induction of diabetes (Fig. 1C and D, Fig. S2). These results collectively suggest that catalase prevents diabetes‐induced myocardial pathology, which is consistent with our previously reported data 2.

**Figure 2:**
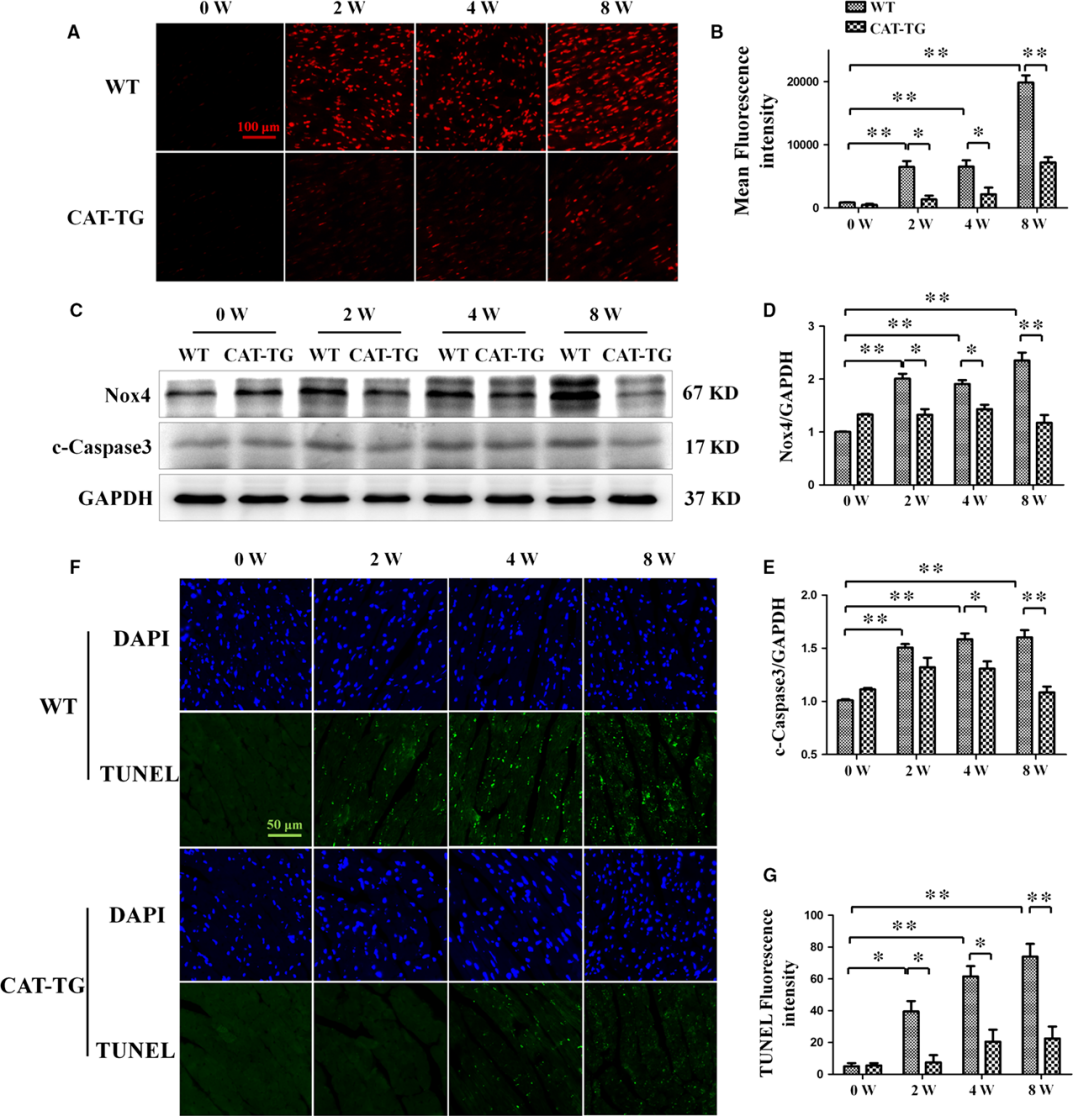
Cardiac catalase overexpression ameliorates diabetes‐induced ROS accumulation and apoptosis. WT and CAT‐TG diabetic mice were analysed 0, 2, 4 and 8 weeks after the induction of diabetes. Dihydroethidine staining was used to detect ROS (**A**) and the resulting fluorescence was measured semi‐quantitatively (**B**). Nox4 and cleaved caspase‐3 expression were evaluated by Western blot, with GAPDH as a loading control (**C**). The band densities of Nox4 (**D**) and cleaved caspase‐3 (**E**) were semi‐quantitatively analysed. The extent of cell death was determined by TUNEL staining (**F** and **G**). Dihydroethidine staining magnification: ×200; bars = 100 μm. Values are mean ± S.E.M. Each group included six mice. *P<0.05, **P<0.01.

Because previous reports suggest that the accumulation of ROS in diabetic mice leads to cardiac hypertrophy 25, 26, we next compared the levels of ROS in myocardial tissues of WT and CAT‐TG diabetic mice. ROS accumulation was elevated in the hearts of WT diabetic mice, while overexpression of the ROS scavenger catalase significantly decreased diabetes‐induced ROS accumulation (Fig. 2A and B). Furthermore, the expression of NADPH oxidase (Nox), which generates superoxide, was up‐regulated in WT diabetic mice, but dramatically suppressed in CAT‐TG diabetic mice (Fig. 2C and D). Catalase overexpression also prevented the diabetes‐induced increase in levels of the apoptotic marker cleaved caspase‐3, especially at the 8 week time‐point (Fig. 2C and E). Finally, TUNEL analysis further confirmed that apoptosis was significantly suppressed in CAT‐TG (Data S1) diabetic mice 2, 4 and 8 weeks after STZ injection (Fig. 2F and G).

### Catalase inhibited diabetes‐induced up‐regulation of autophagy

**Figure 3:**
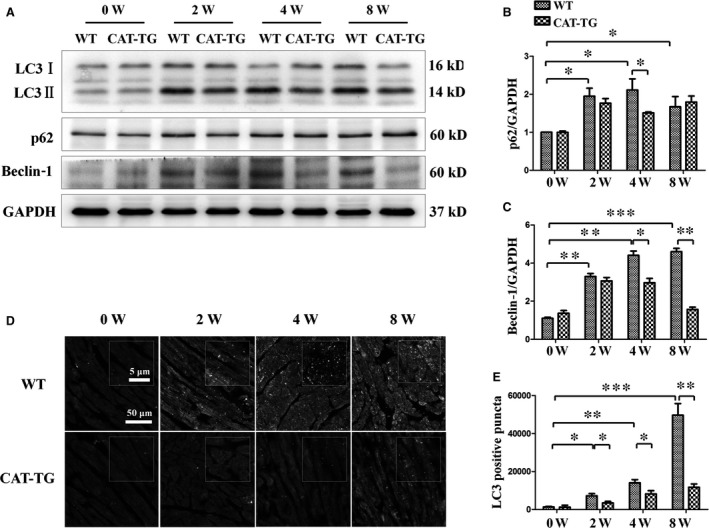
Cardiac catalase overexpression ameliorates diabetes‐induced autophagy. Western blots showing protein levels of LC3‐II, p62 and beclin‐1, with GAPDH as a loading control (**A**). The band densities of p62 (**B**) and cleaved caspase‐3 (**C**) were semi‐quantitatively analysed. The presence of active autophagy was assessed in hearts by immunofluorescent detection of LC3‐II (**D**), and the LC3 dots were semi‐quantitatively analysed (**E**). LC3 fluorography magnification: ×60; bars = 50 μm. Values are mean ± S.E.M. Each group included six mice. *P<0.05, **P<0.01 and ***P<0.001.

ROS accumulation is thought to be the main reason why autophagy is dysregulated in DCM 27, thus, we evaluated changes in autophagy in mice. In WT diabetic mice hearts, expression of the autophagy markers LC3‐II and beclin‐1 was increased, but this effect was blunted in CAT‐TG diabetic mice, especially at 4 and 8 week time‐points (Fig. 3A and C). However, the phosphorylation level of beclin‐1 was similar between WT and CAT‐TG diabetic mice at 0, 2, 4 and 8 weeks (Fig. S3). We also evaluated the effect of diabetes on cardiac autophagy by immunofluorescence staining for LC3‐II in transected hearts. Consistent with the above findings, autophagy flux was significantly higher in diabetic mice at 2 weeks when the mice were administered bafilomycin A1 to block LC3‐II degradation (Fig. S4). After several more weeks, the number of autophagy puncta increased, and catalase overexpression inhibited diabetes‐induced LC3‐II expression (Fig. 3D and E). These results indicate that autophagy was regulated in diabetic myocardial tissues, but not in CAT‐TG diabetic hearts.

**Figure 4:**
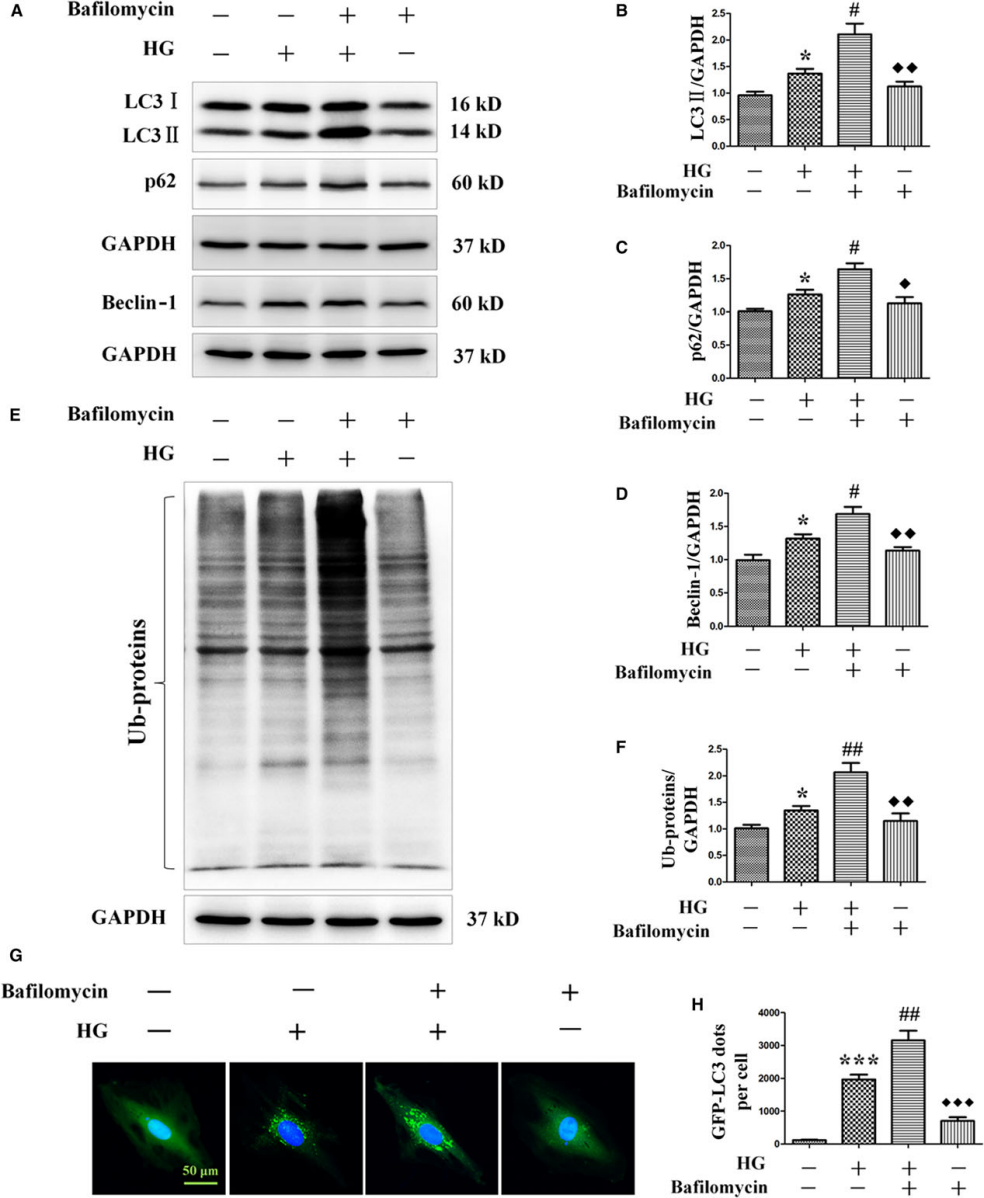
Effects of bafilomycin on the levels of LC3‐II, p62, beclin‐1 and ubiquitinated proteins, and autophagy flux in H9c2 myocardial cells incubated in high‐glucose media. H9c2 myocardial cells were incubated in high‐glucose media (33 mM) for 24 hrs in the absence or presence of the lysosome inhibitor bafilomycin (50 nM). Protein levels of LC3‐II, p62, beclin‐1 and ubiquitinated proteins were detected by Western blot (**A** and **E**). LC3‐II (**B**), p62 (**C**), beclin‐1 (**D**) and ubiquitinated proteins (**F**) levels were normalized with GAPDH protein levels. Autophagy flux was determined by detecting LC3 immunofluorescence dots, and LC3 dots were semi‐quantitatively analysed (**G** and **H**). LC3 fluorography magnification: ×60; bars = 50 μm. Values are mean ± S.E.M. *P<0.05 and ***P<0.001 *versus* Control; #*P*<0.05, ##*P*<0.01 *versus* High glucose; ◆ *P*<0.05, ◆◆ *P*<0.01 and ◆◆◆ *P*<0.001 *versus* High glucose+bafilomycin.

Autophagy is a dynamic process consisting of several stages, such as initiation and degradation 28, 29. To determine the contribution of each stage to autophagy, H9c2 cells were exposed to high‐glucose environment with or without bafilomycin, a lysosome inhibitor that blocks lysosomal degradation and facilitates the measurement of autophagic flux in the heart. We observed that high‐glucose‐induced up‐regulation of autophagy flux was increased by bafilomycin treatment, as shown by the increase in the expression of autophagy marker proteins LC3‐II and beclin‐1 (Fig. 4A,B and D). At the same time, bafilomycin treatment further increased the levels of p62 and ubiquitinated proteins induced by high‐glucose treatment (Fig. 4A,C,E and F). In addition, we confirmed the role of high glucose in autophagy activation by transfecting cardiomyocytes with an adenovirus expressing LC3 fused to green fluorescent protein (GFP‐LC3). Active autophagic flux was detected in the presence of high glucose as shown by the increase in the number of GFP‐LC3 dots in the presence of bafilomycin (Fig. 4G and H). These results suggested that high‐glucose‐induced up‐regulation of autophagy not only increased autophagic flux, but also partly inhibited the degradation of autophagosomes.

### Dysregulation of autophagy leads to apoptosis in diabetic mice

**Figure 5:**
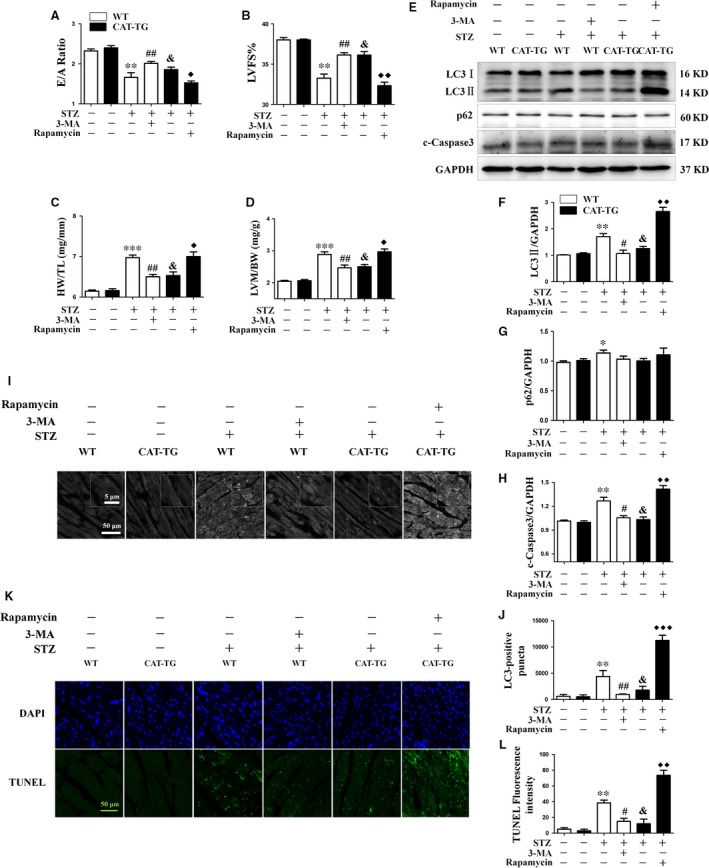
Dysregulation of autophagy leads to apoptosis in diabetic mice. WT and CAT‐TG mice were injected intraperitoneally with normal saline (WT control and CAT‐TG control group), a single dose of STZ (WT diabetes control and CAT‐TG diabetes control group), a single dose of STZ followed by administration of 3‐MA to WT mice (WT diabetes + 3‐MA group), or a single dose of STZ, followed by administration of rapamycin to CAT‐TG mice (CAT‐TG diabetes + rapamycin group). Echocardiography data from six mice per group: E/A ratio (**A**), cardiac left ventricular fractional shortening (LVFS%) (**B**), LV mass per unit body mass (LVM/BW) (**C**) and heart mass to tibial length (HW/TL) (**D**) ratios were measured. LC3‐II, p62 and cleaved caspase‐3 protein expression were evaluated by Western blot, with GAPDH as a loading control (**E**). The band densities of LC3‐II (**F**), p62 (**G**) and cleaved caspase‐3 (**H**) were semi‐quantitatively analysed. Cardiac autophagy was assessed by detection of LC3 dots by immunofluorescence (**I**), and the LC3 dots were semi‐quantitatively analysed (**J**). The degree of cardiac apoptosis was assessed by TUNEL staining (**K**), followed by semi‐quantitative analysis (**L**). LC3 fluorography magnification and TUNEL magnification: ×60; bars = 50 μm. Values are mean ± S.E.M. Each group included six mice. *P<0.05, **P<0.01 and ***P<0.001 *versus* WT Control; #*P*<0.05, ##*P*<0.01 *versus* WT Diabetes; &*P*<0.05 *versus* CAT‐TG Control; ◆*P*<0.05, ◆◆*P*<0.01 and ◆◆◆*P*<0.001 *versus* CAT‐TG Diabetes.

Having shown that diabetes induces cardiac autophagy, we next evaluated the exact role of autophagy in the diabetic myocardium *in vivo*. We injected the autophagy inhibitor 3‐MA and the autophagy activator rapamycin into WT diabetic mice and CAT‐TG diabetic mice, respectively, for up to 8 weeks after the induction of diabetes. As expected, 3‐MA and rapamycin were effective in decreasing the phosphorylation levels of Akt and mTOR, respectively (Fig. S5). Echocardiography showed that 3‐MA partially reversed the diabetes‐induced reductions in cardiac function, whereas the autophagy activator rapamycin impaired the protective effect of catalase overexpression on the diabetic heart. This further aggravated the abnormal cardiac function, indicated by reduced E/A ratio and LVFS% and increases in HW/TL and LVM/BW (Fig. 5A–D). Western blot analysis showed that LC3‐II expression was markedly lower in WT diabetic mice injected with 3‐MA, but was significantly higher in CAT‐TG diabetic mice injected with rapamycin (Fig. 5E and F). Similarly, a larger number of LC3‐II‐positive puncta was observed in CAT‐TG diabetic mice injected with rapamycin, but not in WT diabetic mice injected with 3‐MA (Fig. 5I and J). Therefore, our results demonstrate that 3‐MA, as well as catalase, suppresses autophagy in diabetic mice, but by contrast, rapamycin increases the expression of autophagy‐associated proteins, even when catalase is overexpressed in mice.

Next, we evaluated the effect of autophagy on diabetes‐associated cardiac apoptosis by measuring expression of cleaved caspase‐3. Western blot analysis showed larger amounts of cleaved caspase‐3 in the pro‐autophagic group than in the autophagy inhibited group, implying a higher level of cardiac apoptosis (Fig. 5E and H). In addition, the results of TUNEL staining were consistent with the echocardiography and cleaved caspase‐3 expression data (Fig. 5K and M). These findings indicate that activation of autophagy in diabetes aggravates DCM.

### Catalase overexpression inhibits nuclear translocation of p65 and prevents beclin‐1 up‐regulation in the hearts of diabetic mice

**Figure 6:**
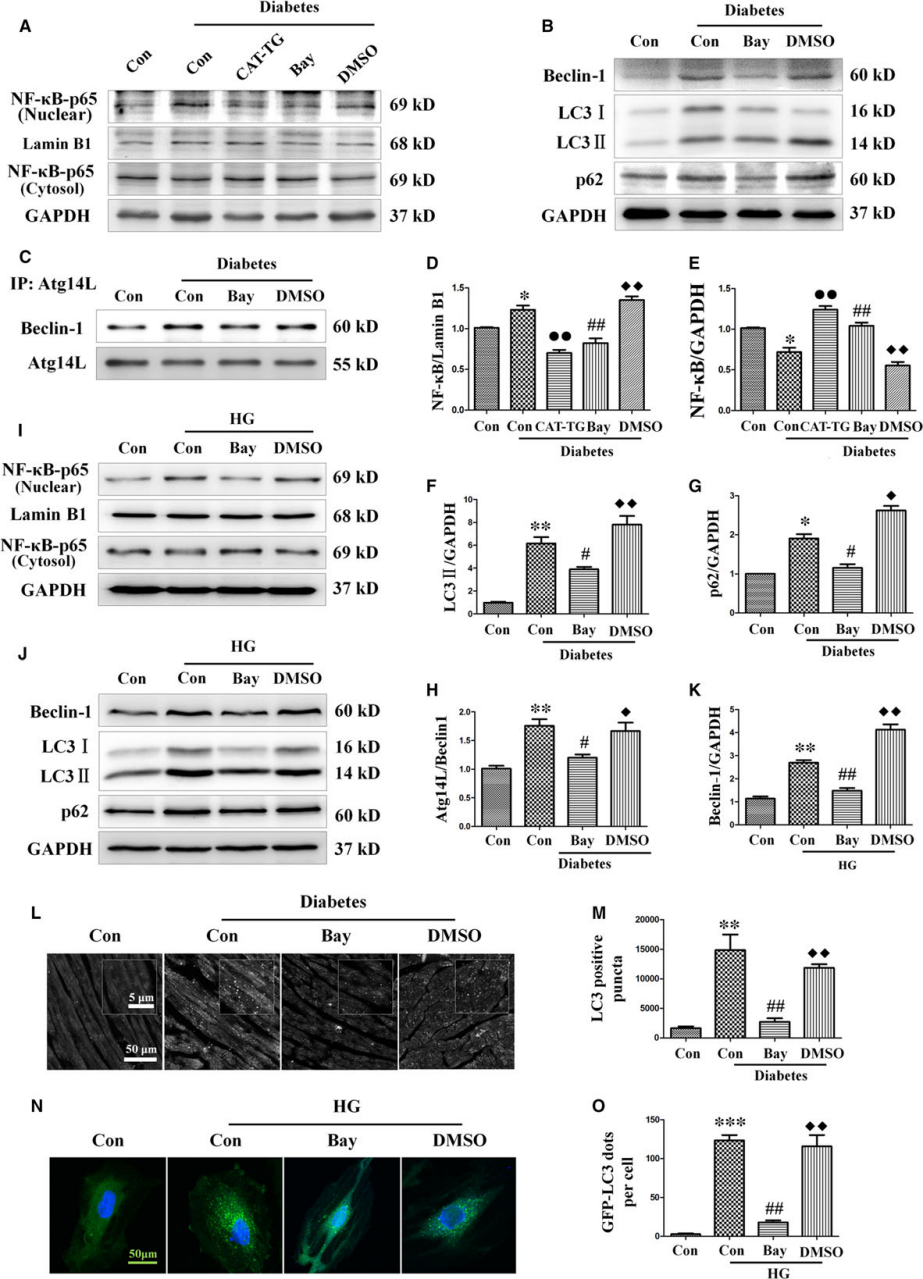
Catalase inhibits nuclear translocation of NF‐κB and treatment with the NF‐κB inhibitor Bay11‐7082 ameliorates diabetes‐induced autophagy. WT mice were injected intraperitoneally with sodium citrate buffer (control group), a single dose of STZ (diabetes control group), a single dose of STZ followed by treatment with Bay11‐7082 (diabetes + Bay11‐7082 group) or a single dose of STZ followed by administration of DMSO (vehicle) (diabetes + DMSO group), and CAT‐TG mice were injected intraperitoneally with a single dose of STZ (CAT‐TG diabetes group). Nuclear and cytosolic protein extracts were prepared, and the expression of NF‐κB was assessed by Western blot analysis (**A**) and semi‐quantitatively analysed, with lamin B1 (**D**) and GAPDH (**E**) being used as loading controls for nuclear and cytosolic protein, respectively. Expression of beclin‐1, LC3‐II and p62 was assessed by Western blot analysis with GAPDH as a loading control (**B**). The band densities of LC3‐II (**F**) and p62 (**G**) were semi‐quantitatively analysed. The binding of Atg14L to beclin‐1 was assessed by co‐immunoprecipitation (**C**) and semi‐quantitatively analysed (**H**). H9c2 myocardial cells were pre‐treated with Bay11‐7082 or vehicle (DMSO) for 10 min. and then incubated in a high‐glucose concentration (33 mM) for 24 hrs. p65 (nuclear and cytosolic fractions) (**I**), beclin‐1, LC3‐II and p62 (**J**) were assessed using Western blot. The band density of beclin‐1 (**K**) was semi‐quantitatively analysed. Mice hearts (**L**) and GFP‐LC3 adenovirus transfected cells (**N**) were used to semi‐quantitatively evaluate autophagy by the detection of LC3 immunofluorescence dots (**M** and **O**, respectively). LC3 fluorography magnification: ×60; bars = 50 μm. Values are mean ± S.E.M. Each group included six mice. *In vivo* results, *P<0.05, **P<0.01 and ***P<0.001 *versus* WT Control; #*P*<0.05, ##*P*<0.01 *versus* WT Diabetes; ◆*P*<0.05, ◆◆*P*<0.01 *versus* WT Diabetes+Bay11‐7082; ●*P*<0.05, ●●*P*<0.01 *versus* WT Diabetes. *In vitro* results, **P<0.01 and ***P<0.001 *versus* Control; ##*P*<0.01 *versus* High glucose; ◆◆*P*<0.01 *versus* High glucose +Bay11‐7082.

Because ROS accumulation results in the phosphorylation of IκB and the activation of NF‐κB signalling during the development of DCM, we next evaluated the nuclear localization of the p65 subunit of NF‐κB 2, 16. Western blot showed that levels of nuclear p65 (Fig. 6A,D and E) and the autophagy protein beclin‐1 (Fig. 3A) were markedly increased in diabetic hearts. Furthermore, the diabetes‐induced increases in nuclear translocation of p65 and beclin‐1 expression were inhibited by catalase overexpression.

### Inhibition of NF‐κB ameliorates diabetes‐associated *BECN1* up‐regulation and autophagy‐mediated cardiac dysfunction

Diabetes‐induced ROS accumulation has been associated with the activation of NF‐κB and autophagy. However, the nature of the mechanistic relationship between these pathological features of the diabetic mice heart is unclear. To analyse the importance of NF‐κB for the initiation and progression of autophagy, the NF‐κB inhibitor Bay11‐7082 was injected into WT diabetic mice for up to 5 weeks, starting 3 weeks after the induction of diabetes. Echocardiography showed that Bay11‐7082 significantly prevented diabetes‐induced cardiac function injury (Table S2). In addition, Bay11‐7082 suppressed nuclear translocation of p65 (Fig. 6A,D and E) and alleviated diabetes‐induced up‐regulation of beclin‐1, LC3‐II and p62 (Fig. 6B,F and G).

Because the beclin‐1‐Vps34‐Atg14L complex is essential for autophagosome formation 30, we investigated the possibility that an NF‐κB‐mediated increase in the binding of beclin‐1 to Atg14L might occur during the formation of the beclin‐1‐Vps34‐Atg14L complex. In WT mice, diabetes significantly increased the binding of beclin‐1 to Atg14L, suggesting that the cardiac levels of the beclin‐1‐Vps34‐Atg14L complex were elevated. By contrast, Bay11‐7082 markedly reduced the level of beclin‐1 binding to Atg14L (Fig. 6C and H). Quantification of the number of LC3‐II‐positive puncta by immunofluorescence also suggested that Bay11‐7082 treatment significantly alleviated the diabetes‐induced increase in autophagy (Fig. 6L and M).

To evaluate the role of NF‐κB in autophagy *in vitro*, we treated H9c2 myocardial cells with or without Bay11‐7082 in medium containing a high concentration of glucose. The results showed that Bay11‐7082 significantly inhibited the high‐glucose‐induced nuclear translocation of p65 (Fig. 6I). In addition, Bay11‐7082 markedly reduced the expression of the key autophagy proteins beclin‐1, LC3‐II and p62, which were up‐regulated in the presence of high‐glucose concentrations (Fig. 6J and K). Finally, infection of H9c2 cells with GFP‐LC3 adenovirus further confirmed that Bay11‐7082 inhibited high‐glucose‐induced activation of autophagy in GFP‐LC3 puncta (Fig. 6N and O).

**Figure 7:**
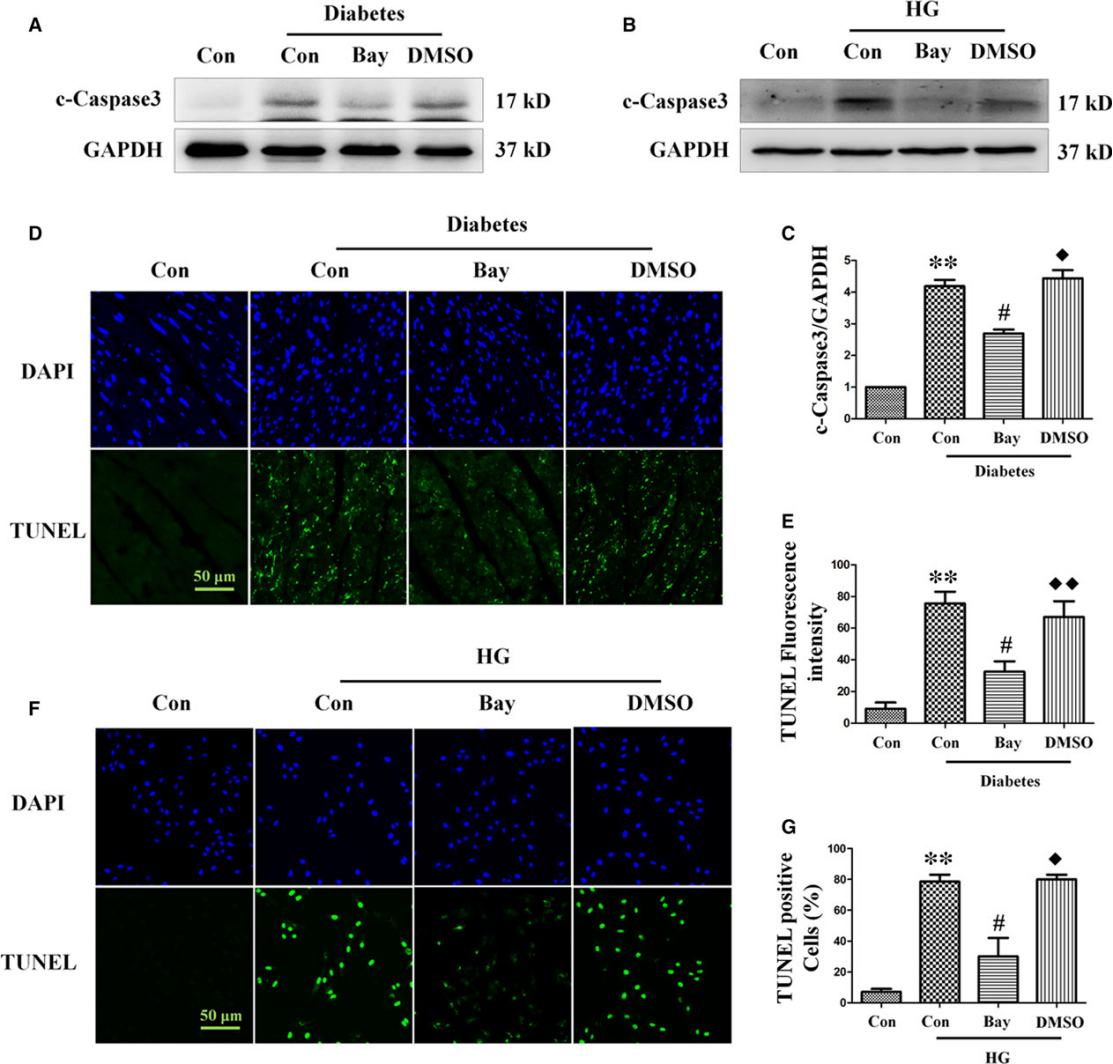
Treatment with the NF‐κB inhibitor Bay11‐7082 ameliorates diabetes‐induced apoptosis. Expression of cleaved caspase‐3 was assessed by Western blot analysis of diabetic heart tissue (**A**) and myocardial cells (**B**). Band densities were semi‐quantitatively analysed (**C**). The degree of apoptosis in heart tissue (**D**) and H9c2 cells (**F**) was assessed by TUNEL staining, followed by semi‐quantitative analysis (**E** and **G**, respectively). TUNEL magnification: ×60; bars = 50 μm. Values are mean ± S.E.M. Each group included six mice. *In vivo* results, **P<0.01 *versus* WT Control; #*P*<0.05, ##*P*<0.01 *versus* WT Diabetes; ◆*P*<0.05, ◆◆*P*<0.01 *versus* WT Diabetes+Bay11‐7082. *In vitro* results, **P<0.01 *versus* Control; #*P*<0.05 *versus* High glucose; ◆*P*<0.05 *versus* High glucose +Bay11‐7082.

To confirm the role of NF‐κB activation in diabetes‐induced cardiac apoptosis, we studied the effect of Bay11‐7082 on diabetic mice hearts and high‐glucose‐treated H9c2 cells. As expected, Bay11‐7082 significantly attenuated high‐glucose‐induced apoptosis both *in vitro* and *in vivo*, as demonstrated by lower cleaved caspase‐3 expression (Fig. 7A–C) and fewer TUNEL‐positive cells (Fig. 7D–G).

**Figure 8:**
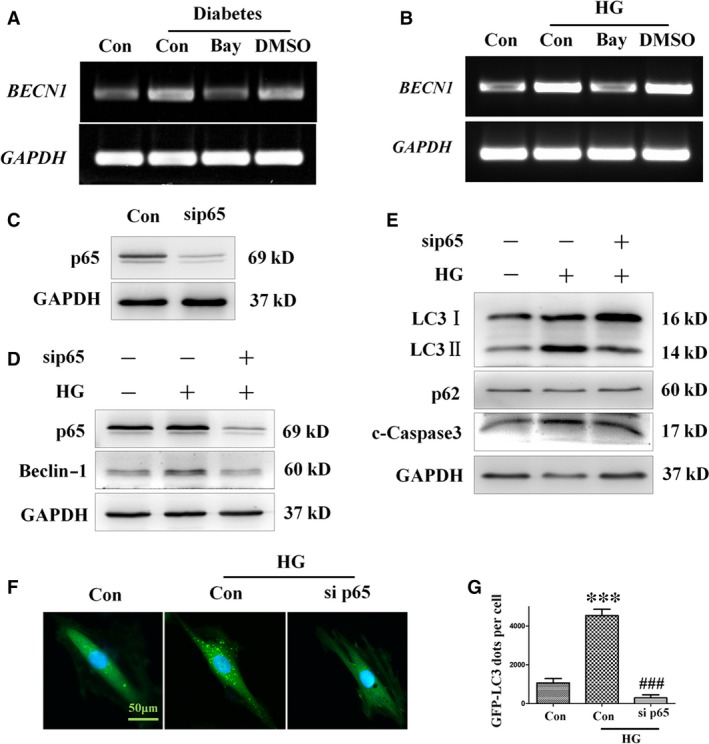
Treatment with the NF‐κB inhibitor Bay11‐7082 ameliorates autophagy and apoptosis in H9c2 cells incubated in high‐glucose medium. H9c2 myocardial cells were pre‐treated with Bay11‐7082 or vehicle (DMSO) for 10 min. and then incubated in high‐ glucose medium (33 mM) for 24 hrs. RT‐PCR analysis of *BECN1* expression in mice (**A**) and H9c2 cells (**B**). H9c2 myocardial cells were transfected with 40 nM p65 siRNA or 40 nM non‐specific (Data S1) control siRNA for 8 hrs and then incubated in 33 mM glucose medium for 24 hrs. Expression of p65 was assessed by Western blot analysis (**C**). The protein levels of p65, beclin‐1 (**D**), LC3‐II, p62 and cleaved caspase‐3 (**E**) were analysed by Western blot analysis. H9c2 cells infected with GFP‐LC3 adenovirus were transfected with 40 nM p65 siRNA or 40 nM non‐specific control siRNA for 8 hrs, and then incubated in high‐ glucose (33 mM) medium for 24 hrs. Autophagy in H9c2 cells transfected with siRNA targeting p65 was assessed by detection of GFP dots (**F**), which were semi‐quantitatively analysed (**G**). GFP‐LC3 fluorography magnification: ×60; bars = 50 μm. Values are mean ± S.E.M. Each group included six mice. ***P<0.001 *versus* Control; ###*P*<0.001 *versus* High glucose.

To further elucidate the relationship between NF‐κB and autophagy, we examined the effect of Bay11‐7082 on the expression of *BECN1* both *in vitro* and *in vivo*. As shown in Figure 8A and B, Bay11‐7082 greatly reduced the expression of *BECN1* both in diabetic mice hearts and high‐glucose‐treated H9c2 cells.

Because the activation of NF‐κB is associated with increased nuclear translocation of p65 during diabetes, the role of NF‐κB activation in autophagy dysfunction was further investigated by silencing p65 expression. To this end, H9c2 cells were transfected with p65‐specific siRNA (sip65), which resulted in a significant decrease in p65 expression (Fig. 8C). Transfection of sip65 dramatically suppressed the high‐glucose‐induced up‐regulation of beclin‐1, LC3‐II and cleaved caspase‐3 (Fig. 8D and E). Moreover, adenovirus‐mediated transfection of GFP‐LC3 H9c2 cells with sip65 attenuated the increase in the number of GFP‐LC3 dots induced by high glucose (Fig. 8F and G), suggesting that expression of p65 is essential for the up‐regulation of autophagy in diabetes, and that the suppression of p65 has a protective effect against DCM.

**Figure 9:**
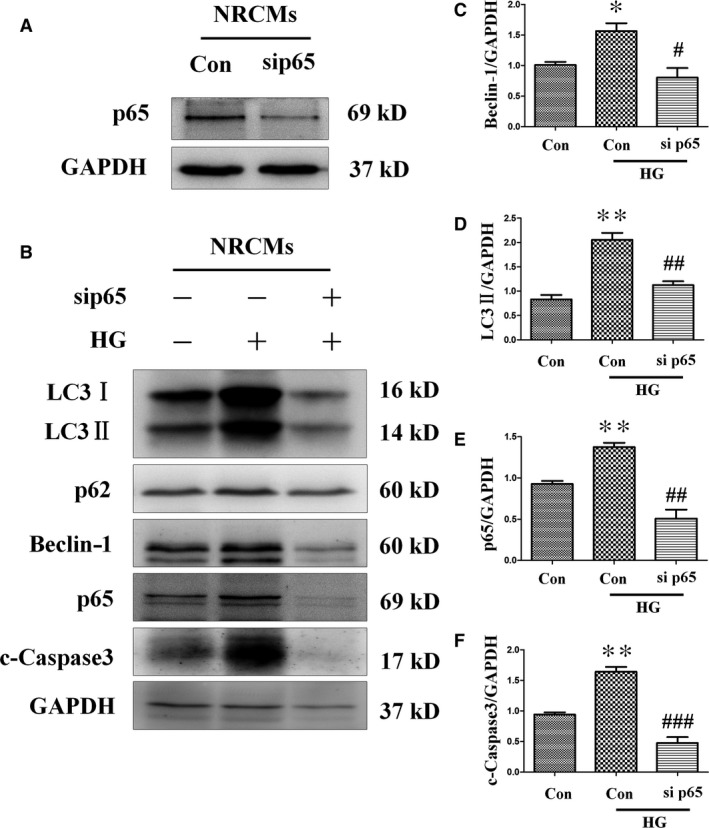
Transfection with sip65 reduced high‐glucose‐induced up‐regulation of autophagy and apoptosis in neonatal rat cardiac myocytes. Cardiomyocytes were infected with 40 nM p65 siRNA or 40 nM non‐specific control siRNA for 8 hrs, and then exposed to 33 mM glucose medium for 24 hrs. Knocking down p65 (**A**,** B** and **E**) blocked autophagy indicated by the differences in the levels of LC3‐II (**B** and **D**) and beclin‐1 (**B** and **C**), which prevented high‐glucose‐induced cell apoptosis, as determined by the level of cleaved caspase‐3 (**F**). Values are mean ± S.E.M. *P<0.05, **P<0.01 *versus* Control; #*P*<0.05, ##*P*<0.01 and ###*P*<0.001 *versus* High glucose.

In addition, to confirm the result obtained with H9c2 cells, which may behave differently to cardiomyocytes, NRCMs were subjected to p65‐specific siRNA treatment, and the effect of NF‐κB on the formation of autophagy was assayed. Consistent with the results obtained in H9c2, the increases in the expression levels of NF‐κB subunit p65 (Fig. 9B and E) and autophagy‐associated proteins beclin‐1 (Fig. 9B and C) and LC3‐II (Fig. 9B and D) induced by high‐glucose treatment were decreased by sip65. Additionally, apoptosis factor cleaved caspase‐3 (Fig. 9B and F) was also up‐regulated by high glucose, but attenuated by sip65. These results indicate that diabetes‐induced autophagy is mediated at least in part by activation of the NF‐κB signalling pathway, and that diabetes‐induced autophagy can be effectively decreased by catalase overexpression.

## Discussion

The data herein establish that hyperglycaemia can activate autophagy, and that a maladaptive autophagic response is involved in diabetes‐induced cardiomyocyte injury. Specifically, intraperitoneal injection of the autophagy inhibitor 3‐MA attenuated diabetes‐induced cardiomyocyte death and cardiac dysfunction, while conversely, activation of autophagy by intraperitoneal injection of rapamycin further aggravated the deleterious effects of diabetes. This implies that uncontrolled autophagy may worsen diabetes‐induced cardiomyopathy. Diabetes‐induced autophagy was markedly suppressed in diabetic mice with cardiac overexpression of catalase, and this was associated with alleviation of diabetes‐induced cardiac apoptosis, and myocardial hypertrophy and dysfunction.

SQSTM1/p62 is a polyubiquitin‐binding protein that is degraded by autophagy 31. Thus, accumulation of p62 can be used to demonstrate inhibition of autophagic degradation in mice with DCM. In this study, we found that the level of p62 was increased in high‐glucose‐treated H9c2 cells, indicating inhibition of autophagic degradation in these cells. Moreover, the p62 level was further increased by bafilomycin in high‐glucose‐treated cells.

It is widely believed that catalase activation can reduce intracellular ROS and thereby inhibit oxidative damage, thus preserving normal cardiac function in DCM 2. Previous studies have investigated the role of catalase in the generation of inflammation as part of the pathogenesis of various diseases 32. In this study, we demonstrated that the NF‐κB family member p65 is important for the regulation of autophagy in diabetes, and that its effects are similar to those observed after the activation of NF‐κB signalling, such as the promotion of either cell death or cell survival, depending on the specific cellular context and the genetic background.

The interaction between p65 and the pro‐autophagy gene *BECN1* regulates the switch between inflammation and autophagy. In addition, beclin‐1 is a component of the class III phosphoinositide 3‐kinase complex that is required for the formation of autophagic vesicles, which it achieves *via* the recruitment of activators of autophagy. This is corroborated by reports showing the inhibition of beclin‐1 expression prevents the induction of autophagy 33.

Interestingly, our experiments showed that both catalase and the NF‐κB inhibitor Bay11‐7082 suppressed diabetes‐induced up‐regulation of the autophagy proteins beclin‐1 and LC3‐II, raising the possibility that the nuclear translocation of p65 may modulate the transcription of *BECN1*, resulting in the subsequent dysregulation of autophagy in the diabetic mice heart. Consistent with this, we found that the activation of NF‐κB signalling and the functional interaction between NF‐κB and *BECN1* play critical roles in the process of autophagy in DCM. We showed that nuclear translocation of p65 in cardiomyocytes, rather than a change in its phosphorylation level, increased the expression of beclin‐1, resulting in dysregulation of autophagy and cardiomyopathy in diabetic mice. These findings point to a novel molecular mechanism underlying the development of cardiac pathology in diabetic patients, especially as altered cardiac autophagy is an important qualitative change during the progression of cardiac myopathy in diabetic mice. Furthermore, catalase inhibits the accumulation of ROS, which in turn activates NF‐κB signalling, resulting in excessive autophagy and protection of the myocardium against damage. These findings may lead to novel approaches for the prevention or treatment of cardiac myopathy, which would improve the quality of life of diabetic patients.

In summary, our findings demonstrate that the protective effects of catalase on DCM can be attributed mainly to its negative effect on NF‐κB signalling and the resultant alleviation of autophagy. Moreover, we revealed that oxidative stress, as a key component in the development of DCM, dramatically promoted the translocation of p65 to the nucleus where it contributed to the transcription of *BECN1*. However, little is known about the p65 binding site in the *BECN1* gene. Whether this interaction plays a critical role in diabetes‐induced myocardial pathology requires further investigation using a diabetic model with appropriate mutations in the *BECN1* promoter.

## Conflict of interest

The authors confirm that there is no conflict of interest.

## Supporting information


**Data S1.** Supplementary methods.
**Table S1.** Echocardiographic findings in WT and CAT‐TG mice injected with sodium citrate buffer (WT Control and CAT‐TG Control group) or STZ (WT Diabetes and CAT‐TG Diabetes group) at 0, 2, 4, and 8 weeks.
**Table S2.** Transthoracic echocardiography was performed in WT mice after administration of sodium citrate buffer (control group), a single dose of STZ (diabetes control group), a single dose of STZ followed by treatment with Bay11‐7082 (diabetes + Bay11‐7082 group), or a single dose of STZ followed by administration of DMSO (vehicle) (diabetes + DMSO group).
**Figure S1.** The levels of blood glucose in control and diabetes WT and CAT‐TG were analyzed 0, 2, 4 and 8 weeks after STZ injection.
**Figure S2.** The histopathology of heart tissues was assessed by staining tissues with hematoxylin and eosin (HE) and Sirius Red (A), and by examining them under a light microscope. Tissues stained with Sirius Red (B) and HE (C) were semi‐quantitatively analyzed as described in Materials and Methods. Magnification, ×200; bars = 100 μm. Values are mean ± SEM, *n* = 6–8 per group. *P<0.05, **P<0.01 and ***P<0.001 vs. WT Control; #*P*<0.05 and ##*P*<0.01vs. CAT‐TG Control; ◆*P*<0.05 and ◆◆*P*<0.01 vs. WT Diabetes.
**Figure S3.** WT and CAT‐TG diabetic mice were analyzed 0, 2, 4 and 8 weeks after the induction of diabetes.
**Figure S4.** WT and CAT‐TG mice were evaluated at 2 weeks after induction of diabetes.
**Figure S5.** The levels of expression of phosphorylated Akt and mTOR were detected by western blot analysis of WT diabetic mice treated with 3‐MA and CAT‐TG diabetic mice treated with rapamycin, respectively. *n* = 6–8 per group.
