# Supplementary material for: Catalase ameliorates diabetes‐induced cardiac injury through reduced p65/RelA‐ mediated transcription of BECN1
Source: J Cell Mol Med. 2017 Jun 23;21(12):3420–34. doi: 10.1111/jcmm.13252 (PMC5706580; doi:10.1111/jcmm.13252)
Supplement: Supplementary file 1 — Data S1. Supplementary methods. Table S1. Echocardiographic findings in WT and CAT‐TG mice injected with sodium citrate buffer (WT Control and CAT‐TG Control group) or STZ (WT Diabetes and CAT‐TG Diabetes group) at 0, 2, 4, and 8 weeks. Table S2. Transthoracic echocardiography was performed in WT mice after administration of sodium citrate buffer (control group), a single dose of STZ (diabetes control group), a single dose of STZ followed by treatment with Bay11‐7082 (diabetes + Bay11‐7082 group), or a single dose of STZ followed by administration of DMSO (vehicle) (diabetes + DMSO group). Figure S1. The levels of blood glucose in control and diabetes WT and CAT‐TG were analyzed 0, 2, 4 and 8 weeks after STZ injection. Figure S2. The histopathology of heart tissues was assessed by staining tissues with hematoxylin and eosin (HE) and Sirius Red (A), and by examining them under a light microscope. Tissues stained with Sirius Red (B) and HE (C) were semi‐quantitatively analyzed as described in Materials and Methods. Magnification, ×200; bars = 100 μm. Values are mean ± SEM, n = 6–8 per group. *P<0.05, **P<0.01 and ***P<0.001 vs. WT Control; #P<0.05 and ##P<0.01vs. CAT‐TG Control; ◆P<0.05 and ◆◆P<0.01 vs. WT Diabetes. Figure S3. WT and CAT‐TG diabetic mice were analyzed 0, 2, 4 and 8 weeks after the induction of diabetes. Figure S4. WT and CAT‐TG mice were evaluated at 2 weeks after induction of diabetes. Figure S5. The levels of expression of phosphorylated Akt and mTOR were detected by western blot analysis of WT diabetic mice treated with 3‐MA and CAT‐TG diabetic mice treated with rapamycin, respectively. n = 6–8 per group. [file JCMM-21-3420-s001.docx]

**Supplementary Data**

**to**

**Catalase ameliorates diabetes-induced cardiac injury through reduced p65/RelA- mediated transcription of *BECN1***

Xu Wang ^a,^ *, Youli Tao ^a, b,^ *, Yewei Huang ^a,^ *, Kungao Zhan ^c,^ *, Mei Xue ^a^, Ying Wang ^a^, Dandan Ruan ^d^, Yangzhi Liang ^a^, Xiaozhong Huang ^a^, Jianjun Lin ^e^, Zhiwei Chen ^a^, Lingchun Lv ^f^, Santie Li ^a^, Gen Chen ^a^, Yang Wang ^g^, Ruijie Chen ^c,^ **, Weitao Cong ^a,^ **, Litai Jin ^a,^ **

**Supplementary Methods**

**Echocardiography**

Mice after 0, 2, 4 and 8 weeks of diabetes were detected by echocardiography. Transthoracic echocardiography involved use of the Vevo 770 imaging system equipped with high-resolution transducer (VisualSonics, Toronto, Canada). Mice were anaesthetized with intraperitoneal injection of pentobarbitone sodium 60 mg/kg BW. All measurements were in accordance with the modiﬁed American Society of Echocardiography recommendations. Diastolic transmitral left ventricular inﬂow images were obtained from apical four-chamber views using color ﬂow mapping-guided pulsed-wave Doppler and were used to calculate early (E) and late (atrial, A) peak ﬁlling blood ﬂow velocities [[1](#_ENREF_1)]. A single hind leg was removed to determine tibia length (TL). All measurements represent the mean of at least six consecutive cardiac cycles.

**TUNEL Staining**

Heart tissue was soaked in paraformaldehyde for 24 h, dehydrated with concentration gradient sucrose, embedded in OTC and sectioned at 5 μm. H9c2 cells were grown in coverslips, after reaching 60% of density, pretreated with 2 μM Bay11-7082 or DMSO for 10 min, then incubated with high-glucose (33 mM) for 24 h. H9c2 cells and cardiac apoptotic nuclei were examined with transferase mediated dUTP nick-end labeling (TUNEL) staining using DeadEnd Fluorometric TUNEL System (Promega, Madison, USA). Detailed steps comply with TUNEL staining manufacturer’s instructions.

**RT-PCR**

Total RNA were extracted from heart tissue and cells by use of TRIzol reagent (Invitrogen, Carlsbad, California, USA) and reverse transcribed by using a cDNA reverse transcription kit (Reverse Transcription System, Promega). qRT-PCR was performed, and gene expression was quantiﬁed as described previously [[2](#_ENREF_2)]. The mRNA levels were normalized against that of GAPDH. Gene speciﬁc primer sequences used for qRT-PCR are list as follow, the primers used to amplify the rat *BECN1* gene were 5’-GTGCTCCTGTGGAATGGAAT-3’ (forward) and 5’-GCT GCACACAGTCCAGAAAA-3’ (reverse), mouse *BECN1* gene were 5’-GTACC GACTTGTTCCCTATGG-3’ (forward) and 5’-ACACAGTCCA GAAAAGCTA CC-3’ (reverse). Each experiment was repeated in triplicate.

**Protein extraction**

To separate cytoplasmic protein and nuclear protein, heart tissues were minced into small pieces and homogenized in an ice-cold lysis solution (AR0101-100, Boster, Wuhan, China) by tissue grinder. Cytoplasmic protein and nuclear protein extracts were obtained by using a Nuclear Protein Extraction Kit (KeyGEN, Nanjing, China) according to the manufacturer's instructions. Protein concentrations were measured using Bradford protein assay kits (Bio-Rad, Richmond, CA, USA) [[3](#_ENREF_3)].

**Western blot analysis**

Total protein from heart tissues or cells was extracted by use of ice-cold lysis solution (AR0101-100, Boster, Wuhan, China). Proteins were incubated with the primary antibodies anti-Nox4 (Origene, TA323911), anti-cleaved-caspase3 (Cell signaling, 9661), anti-LC3A/B (Abcam, Ab62721), anti-p62 (Cell signaling, 5114), anti-Beclin-1 (Cell signaling, 3495), anti-NF-κB-p65 (Abcam, Ab7970), anti-GAPDH (Abcam, Ab8245), anti-Lamin B (Cell signaling, 12586), anti-Atg14L (Abcam, Ab139727) and then incubated with goat anti-rabbit or anti-mouse IgG secondary antibodies. Antigen-antibody complexes were visualized by using an electrochemiluminescence (ECL) kit (GE healthcare) and calculated by densitometry with Quantity one. Each assay was repeated at least three times.

**Supplementary references**

1. **Luo T, Kim JK, Chen B, Abdel-Latif A, Kitakaze M, Yan L.** Attenuation of ER stress prevents post-infarction-induced cardiac rupture and remodeling by modulating both cardiac apoptosis and fibrosis. *Chemico-biological interactions*. 2015; 225: 90-8.

2. **Zittermann SI, Issekutz AC.** Basic fibroblast growth factor (bFGF, FGF-2) potentiates leukocyte recruitment to inflammation by enhancing endothelial adhesion molecule expression. *The American journal of pathology*. 2006; 168: 835-46.

3. **Cong W, Zhao T, Zhu Z, Huang B, Ma W, Wang Y, Tan Y, Chakrabarti S, Li X, Jin L, Cai L.** Metallothionein prevents cardiac pathological changes in diabetes by modulating nitration and inactivation of cardiac ATP synthase. *The Journal of nutritional biochemistry*. 2014; 25: 463-74.

**Supplementary Data**

| **Table S1.** | **Biometric and echocardiographic measurement parameters of mice injected STZ** | | | | | |
| --- | --- | --- | --- | --- | --- | --- |
|  | | **WT** | | **CAT-TG** | | |
|  | | **Control** | **Diabetes** | **Control** | **Diabetes** | |
| **E/A Ratio** | | |  |  | |  |
| 0 W | | 2.32±0.05 | 2.31±0.08 | 2.31±0.05 | 2.29±0.05 | |
| 2 W | | 2.24±0.06 | **2.03±0.08*** | 2.26±0.07 | 2.14±0.02 | |
| 4 W | | 2.16±0.06 | **1.74±0.06**** | 2.17±0.05 | **2.06±0.06#** | |
| 8 W | | 2.16±0.04 | **1.59±0.09**** | 2.20±0.07 | **1.99±0.07#◆** | |
| **LVFS%** | | |  |  |  | |
| 0 W | | 36.5±0.5 | 36.0±1.0 | 36.7±0.7 | 36.5±0.5 | |
| 2 W | | 36.5±0.7 | **35.3±0.7*** | 36.4±0.4 | 36.0±0.3 | |
| 4 W | | 36.6±0.4 | **33.9±0.4**** | 36.0±0.7 | **35.5±0.3#** | |
| 8 W | | 36.2±0.8 | **32.0±0.5**** | 36.3±0.3 | **34.6±0.4#◆** | |
| **HW/TL(mg/mm)** | | |  |  |  | |
| 0 W | | 6.14±0.04 | 6.13±0.03 | 6.11±0.07 | 6.14±0.03 | |
| 2 W | | 6.06±0.06 | 6.21±0.04 | 6.11±0.08 | 6.24±0.03 | |
| 4 W | | 6.25±0.05 | **6.52±0.05*** | 6.25±0.05 | **6.24±0.04#** | |
| 8 W | | 6.45±0.05 | **7.04±0.09**** | 6.38±0.08 | **6.60±0.05#◆** | |
| **LVM/BW(mg/g)** | | |  |  |  | |
| 0 W | | 2.08±0.06 | 2.10±0.07 | 2.07±0.07 | 2.08±0.05 | |
| 2 W | | 2.05±0.05 | 2.15±0.05 | 2.10±0.06 | 2.10±0.03 | |
| 4 W | | 2.02±0.04 | **2.35±0.05*** | 2.10±0.04 | 2.24±0.06 | |
| 8 W | | 2.11±0.05 | **3.00±0.10***** | 2.05±0.05 | **2.41±0.06#◆** | |

**Table S1.** [Echocardiographic](javascript:void(0);) findings in WT and CAT-TG mice injected with sodium citrate buffer (WT Control and CAT-TG Control group) or STZ (WT Diabetes and CAT-TG Diabetes group) at 0, 2, 4, and 8 weeks. E/A ratio, cardiac left ventricular fractional shortening (LVFS%), LV mass per unit body mass (LVM/BW) and ratio of heart mass to tibial length (HW/TL) were measured. Values are mean ± SEM, n = 6–8 per group. *****p＜0.05, **********p＜0.01 and *******p＜0.001 vs. WT Control; ^#^p＜0.05 vs. CAT-TG Control; ^◆^p＜0.05 vs. WT Diabetes.

| **Table S2.** | **Biometric and echocardiographic measurements parameters of mice injected with STZ with or without Bay11-7082** | | | | | |
| --- | --- | --- | --- | --- | --- | --- |
|  | | | **Control** | **Diabetes** | | |
|  | | |  | **Control** | **Bay11-7082** | **DMSO** |
| **E/A Ratio** | | | 2.30±0.20 | 1.67±0.13****** | 2.20±0.10# | 1.56±0.13**◆◆** |
| **LVFS%** | | | 35.1±1.1 | 32.3±1.3***** | 34.8±0.8# | 29.5±1.5**◆◆** |
| **HW/TL(mg/mm)** | | | 6.05±0.15 | 6.74±0.16****** | 6.25±0.15# | 6.99±0.21**◆◆** |
| **LVM/BW(mg/g)** | | | 2.09±0.09 | 2.83±0.17***** | 2.35±0.15# | 3.05±0.15**◆◆** |

**Table S2.** Transthoracic echocardiography was performed in WT mice after administration of sodium citrate buffer (control group), a single dose of STZ (diabetes control group), a single dose of STZ followed by treatment with Bay11-7082 (diabetes + Bay11-7082 group), or a single dose of STZ followed by administration of DMSO (vehicle) (diabetes + DMSO group). E/A ratio, cardiac left ventricular fractional shortening (LVFS%), LV mass per unit body mass (LVM/BW) and ratio of heart mass to tibial length (HW/TL) were measured. Values are mean ± SEM, n = 6–8 per group. *****p＜0.05 and ******p＜0.01 vs. Control; ^#^p＜0.05 vs. Diabetes; ^◆◆^p＜0.01 vs. Diabetes + Bay11-7082.


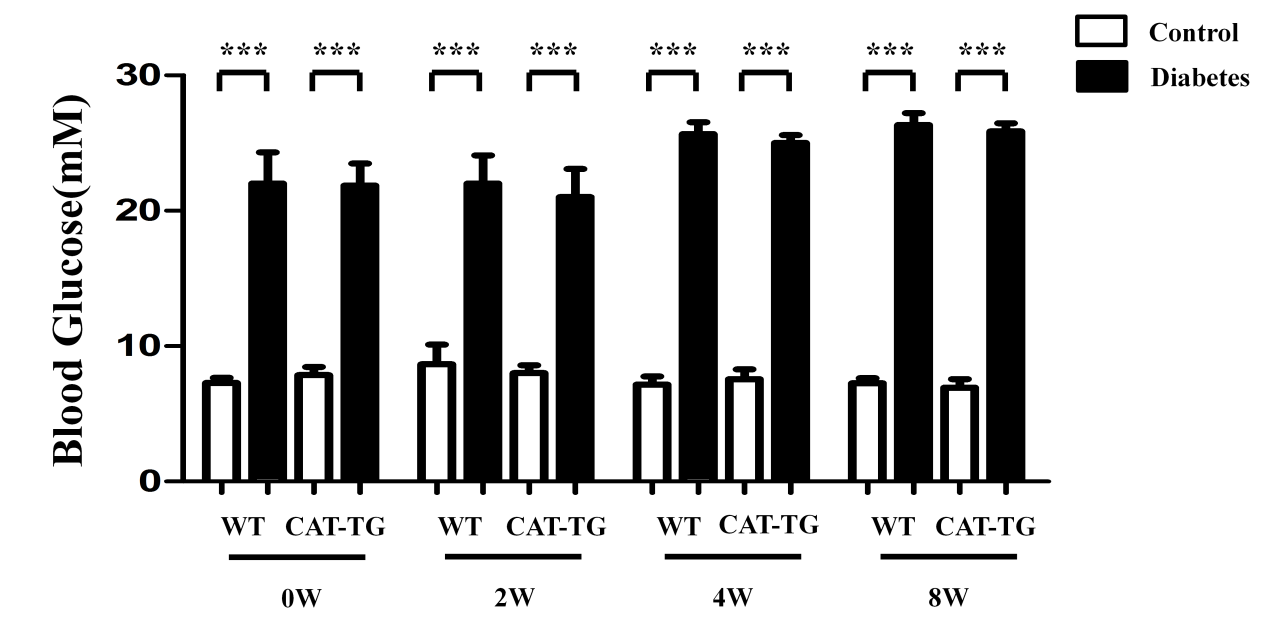


**Figure S1.** The levels of blood glucose in control and diabetes WT and CAT-TG were analyzed 0, 2, 4 and 8 weeks after STZ injection. The levels of blood glucose were increased after 72 h, values are mean ± SEM, n = 6–8 per group. ***p＜0.001


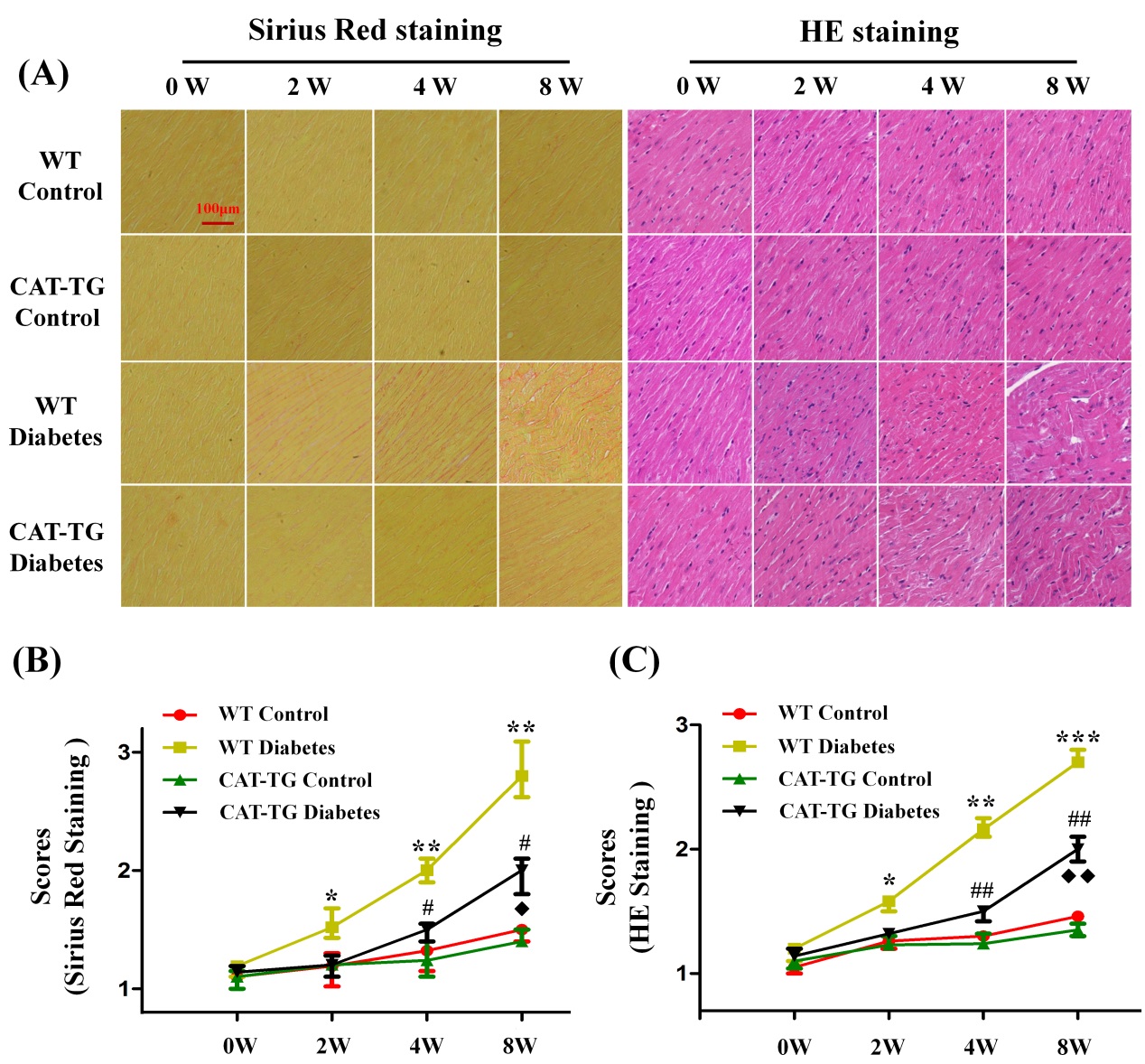


**Figure S2.** The histopathology of heart tissues was assessed by staining tissues with hematoxylin and eosin (HE) and Sirius Red (A), and by examining them under a light microscope. Tissues stained with Sirius Red (B) and HE (C) were semi-quantitatively analyzed as described in Materials and Methods. Magniﬁcation, ×200; bars = 100 μm. Values are mean ± SEM, n = 6–8 per group. *****p＜0.05, ******p＜0.01 and *******p＜0.001 vs. WT Control; ^#^p＜0.05 and ^##^p＜0.01vs. CAT-TG Control; ^◆^p＜0.05 and ^◆◆^p＜0.01 vs. WT Diabetes.

**
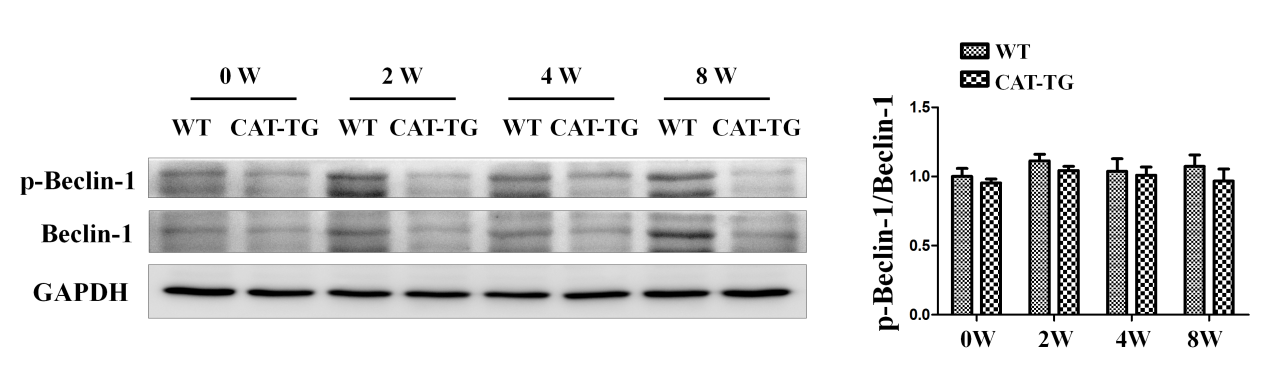
Figure S3.** WT and CAT-TG diabetic mice were analyzed 0, 2, 4 and 8 weeks after the induction of diabetes. Western blots showed the phosphorylation protein level of beclin-1, with beclin-1 as a loading control. The band density of p-Beclin-1 was semi-quantitatively analyzed. n = 6–8 per group.





**Figure S4.** WT and CAT-TG mice were evaluated at 2 weeks after induction of diabetes. Immunofluorescent detection of LC3 dots in heart tissue section, LC3 fluorography magniﬁcation: ×60; bars = 50 μm. Values are mean ± SEM. n = 6–8 per group.





**Figure S5.** The levels of expression of phosphorylated Akt and mTOR were detected by western blot analysis of WT diabetic mice treated with 3-MA and CAT-TG diabetic mice treated with rapamycin, respectively. n = 6–8 per group.
